# Older Adult Perspectives on Integrative Pain Management, Analgesics and Educational Preferences

**DOI:** 10.7759/cureus.80432

**Published:** 2025-03-11

**Authors:** Sophia Sheikh, Taylor Munson, Jennifer Brailsford, Monika Patel, Jason Beneciuk, Robin M Li, Morgan Henson, Natalie Spindle, Megan E Curtis, Phyllis Hendry

**Affiliations:** 1 Emergency Medicine, University of Florida College of Medicine – Jacksonville, Jacksonville, USA; 2 Center for Data Solutions, University of Florida Health, Jacksonville, USA; 3 Neurosciences Institute, Cleveland Clinic Florida, Weston, USA; 4 Physical Therapy, University of Florida, Gainesville, USA; 5 Physical Therapy, Brooks Rehabilitation Hospital, Jacksonville, USA; 6 Pharmacy Education and Practice, University of Florida College of Pharmacy, Gainesville, USA

**Keywords:** integrative medicine, older adult, opioid alternatives, pain management, patient experience

## Abstract

Older adults’ (adults over age 50) preferences for educational programs on integrative pain management have not been adequately explored. Integrative pain management uses a holistic approach combining traditional medical treatments with alternative non-pharmacological practices. Pain-related education targeting older adults is supported by prior research, but to our knowledge older adult perspectives and learning preferences have not been investigated. Our objective was to explore older adult views on integrative pain management, specifically their educational needs and preferences, for patient-centered pain management education. Patients >50 years of age with chronic pain (pain for three months or longer), were eligible for enrollment in virtual focus groups. Audio recordings were transcribed, coded, and analyzed via a mixed inductive-deductive framework approach using ATLAS.ti version 23.0.8.0 (Microsoft, Redmond, WA, USA). Descriptive statistics were performed using Stata 16 (StataCorp., College Station, TX, USA).

There were 16 participants and five themes generated: opioid/analgesic perceptions, integrative pain management, patient-provider relationship, and educational needs/preferences. All participants felt traditional interactions with healthcare providers did not adequately address their educational needs. There were discordant views on opioids, some noted positive impacts on pain and function and others feared addiction and side effects. Subthemes on the patient-provider relationship were identified including misalignment in treatment preferences/goals and communication gaps. Participants preferred virtual programs, incorporating demonstrations, audience interaction, and physical materials. These findings can be used to develop patient-centered educational programs

## Introduction

Approximately 50% of older adults in the United States (U.S.) experienced bothersome pain within the past month [1]. Among the older adult population in the U.S., 15% were prescribed an opioid prescription within the last 12 months and 35% of those receiving an opioid prescription reported opioid prescription misuse [2]. Efforts to decrease opioid use-related risks are rapidly transforming all facets of healthcare (e.g., patient care, clinical operations, finances, legislation, and research) [3]. Developing safe and effective treatment plans is challenging in older adults due to limited pharmacological resources secondary to multiple comorbidities, polypharmacy, and age-related physiologic changes.

An expanding body of literature indicates that integrative strategies may reduce opioid dependence, chronic pain symptoms, and risk for opioid addiction [4-6]. However, few studies have explored the older adult perspective on integrative pain management and use of prescription opioids in treating chronic pain, providing a vital gap in provision of healthcare services for these individuals. Therefore, development of effective educational strategies on integrative pain management practices tailored to the older adult population is needed [7]. In 2019, the National Council on Aging reported, “81% of older adults do not understand safe, effective, and affordable alternatives to reducing pain without prescription opioid medications” [8]. These findings may be attributed to limited evidence on how older adults prefer to learn about integrative pain management options [9].

Aging individuals have existing beliefs about pain and aging, which may negatively affect the perception of pain experience and functional outcomes of the individual [8,9]. For example, some older adults believe that physical activity is unnecessary or potentially harmful [10]. Previous studies indicate that education may be effective in refuting this misinformation and improving health outcomes for this population [11].

Integrative pain management topics incorporating patient education include but are not limited to nutrition, acupuncture, aromatherapy, and cannabis use. Research has shown there is a positive correlation between dietary intake and pain experiences [12]. Diet modifications could include reducing consumption of inflammatory food, while increasing the intake of fruits, vegetables, unsaturated fats and omega-3 fatty acids. Some individuals achieve this diet modification by adapting their diet to a plant-based or Mediterranean diet [13]. Another integrative approach is acupuncture. Recent literature indicates strong evidence acupuncture is effective in managing chronic pain, as well as providing short-term pain relief and mobility improvement [13,14]. Aromatherapy has shown to be successful when used as a postoperative intervention [15]. Cannabis has gained popularity as a non-traditional, and controversial, option for managing pain. A cross-sectional study showed more than half of their study population (N=1,724) used cannabis to manage chronic pain while simultaneously decreasing their use of prescription opioids, non-opioids, and over-the-counter pain medication [16]. Given its popularity and the limited research demonstrating efficacy, additional studies are needed particularly from the patient perspective on the role of cannabis in pain management.

While literature supports a need for more pain-related educational initiatives targeting older adults, to our knowledge none have explored older adult perspectives and learning preferences for receiving education on these topics, particularly those related to integrative pain management. Our primary objective was to explore the perspectives of older adults regarding integrative pain management. Specifically, we aimed to understand their educational needs and learning preferences to develop effective, patient-centered pain management strategies

## Materials and methods

Study design

Focus groups and semi-structured interviews were conducted to better understand integrative pain management experiences and educational needs and preferences of older adults (>/= 50 years old) who experience chronic pain and/or their caregivers. Participants completed a one-hour focus group or semi-structured interview via Zoom Video Communications (Zoom Communications, Inc., San Jose, CA, USA). Participant recruitment occurred in the Northeast Florida region using a convenience sampling methodology (e.g., email through established listservs). A research coordinator was available during normal work hours (e.g., 8 am-10 pm) to answer questions and to assist with the informed consent process. The informed consent process was completed in two steps: (1) interested individuals reviewed the consent and then agreed to leave their contact information (phone, email, other) and (2) research coordinator contacted those who consented to schedule a time to participate in a focus group. Study data were collected and managed in REDCap Electronic Data Capture tools hosted at the University of Florida. Study methods and materials were approved by the University of Florida Institutional Review Board.

Participant recruitment

**Figure 1:**
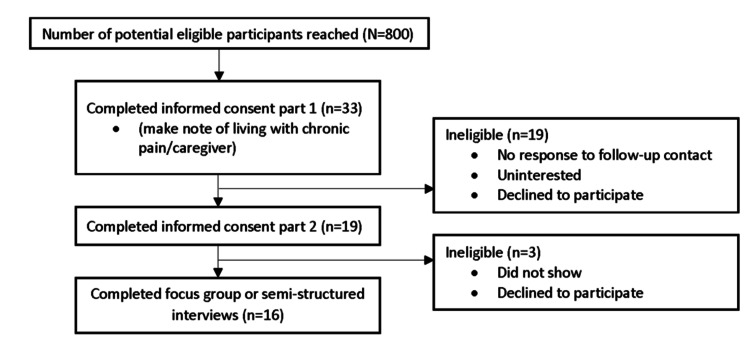
Enrollment Schematic

Adults >/= 50 years old with chronic pain and their caregivers were recruited between August 2020 and December 2021. Eligibility criteria included individuals who experience chronic pain or who served as the primary caregiver for someone experiencing chronic pain and had the ability to access focus groups and semi-structured interviews remotely. All participants were English-speaking and completed two informed consent forms. Of the 800 eligible individuals who were contacted, 33 individuals completed the first informed consent form. Sixteen out of 33 individuals (48%) agreed to take part in a focus group or semi-structured interview, and completed the second informed consent form (see Figure 1).

Data collection strategies

A multi-disciplinary team of stakeholders (a qualitative researcher, a health educator, and clinical pain management experts including physicians, physical therapists, and pharmacists) developed an outline of questions (i.e., discussion guide) based on a priori issues identified through a review of existing literature until a consensus was reached. To our knowledge, stakeholders did not have any association with the care of study participants. A semi-structured discussion guide (see Appendix) was developed to obtain a better understanding of (1) participant satisfaction with their current pain management plan and healthcare provider(s) in managing their pain; (2) impact of pain on their life; (3) attitudes and beliefs toward pain medications (e.g., opioids, and non-opioid analgesics); (4) interest in non-pharmacologic pain management options (e.g., aromatherapy, yoga, physical therapy and nutrition); and (5) participants educational preferences about non-pharmacologic pain management topics (type of setting, materials, structure/format, type of instructor, etc.). A one-hour focus group with three participants and 13 semi-structured interviews were conducted via Zoom Video Communications from October 2020 to February 2021. Sessions were led by facilitators with advanced degrees and training in social science, population health, qualitative methodologies, and communications. Participants were informed that the sessions were confidential and would be audio recorded and transcribed. Audio recordings and transcriptions of the focus groups and semi-structured interviews were reviewed for accuracy and coded by the study team.

Data analysis

Verbatim transcripts were analyzed using a mixed inductive-deductive approach with the Framework Method to manage the generated data [17]. Themes were generated both a priori (as discussed above) and were also allowed to organically emerge by using an open coding approach [17]. Thus, although we had themes a priori that were informed by the literature and outlined in the discussion guide, we analyzed data and developed codes with an open coding approach thereby remaining open to all codes and any new themes that may emerge. Codes that did not fit into the a priori themes within the framework were retained and potentially grouped as an emergent theme [18]. Three members of the research team independently reviewed the transcripts, coded, and met at intervals during the process to discuss emerging codes, develop consensus and identify overarching themes among codes when applying the analytical framework via ATLAS.ti version 23.0.8.0. (Microsoft, Redmond, WA, USA) Thirty-one researchers moved from codes to themes through iterative meetings in which codes were defined, grouped into subthemes, then themes as the team identified relationships in the data.

Analysis proceeded in the seven stages prescribed by this method (Table 1).

**Table 1:** Data Analysis Methods

Transcription	Audio recordings were transcribed using Otter.AI, an automated transcription platform. Transcripts were reviewed for accuracy by the study team.
Familiarization	Study team familiarized themselves with the data by thoroughly reading all transcripts.
Coding	All transcripts were independently coded by NS and JB using open coding. MH and EV coded a sample of transcripts to validate the coding process.
Developing an analytic framework	All researchers met to compare codes and construct an initial coding framework. Coded data were clustered according to subthemes and grouped into themes.
Applying framework	The agreed upon framework was systematically applied to all transcripts using a qualitative data management software, Atlas.ti 8 (Berlin, Germany). The researchers then organized codes which were valuable for addressing the research question into subthemes reflecting a priori themes within the data set. Codes which were relevant to the research question but did not fit into a priori themes were retained for the discovery of emergent themes.
Charting	A spreadsheet was created to generate a matrix of each theme by abstracting, summarizing, and charting data and illustrative quotations for each code within subthemes and then overarching themes.
Interpretation	Thematic analysis was carried out on the managed data set by reviewing the matrices and making connections within and between codes.

Data saturation of codes was achieved once new data collected became redundant from previous data collected and there was consensus across views expressed by respondents [18,19]. This occurred after 14 sessions with 16 respondents. Descriptive statistics of demographic and professional characteristics were performed for the study population using Stata 16 (StataCorp., College Station, TX, USA).

**Figure 2:**
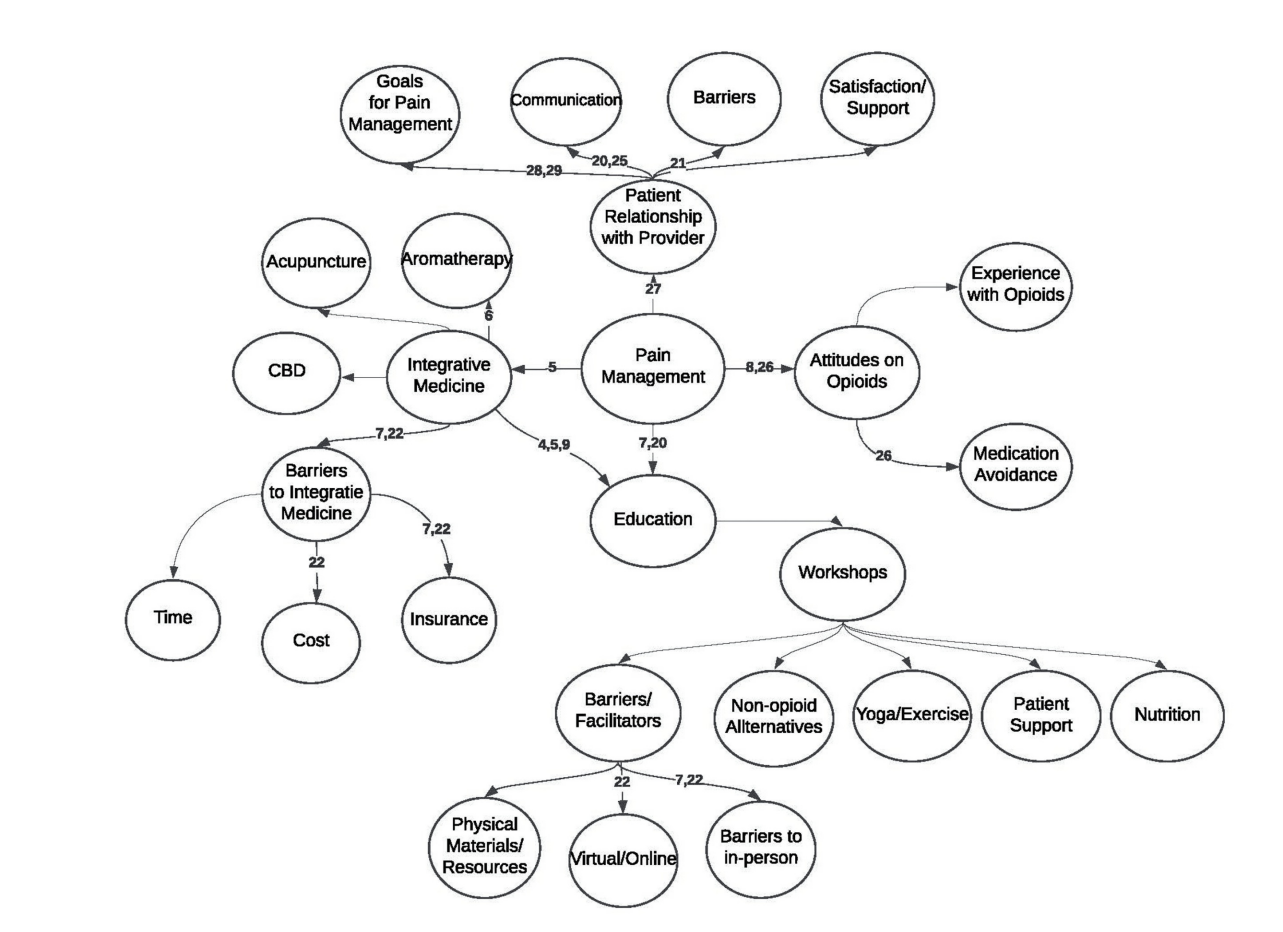
Mind Map of Interconnected Themes The references supporting these connections are noted within the figure [4-9,21-22,25-29] CBD: Cannabidiol

A Mind Map of themes is presented in Figure 2. This Mind Map depicts the structural schema of interconnected themes, sub-themes, and codes presented in the results. It includes references to the literature supporting these connections.

## Results

We conducted one focus group (n=3) and 13 semi-structured interviews, totaling 16 participants (Table 2).

**Table 2:** Participant Demographic Characteristics (N=16,%)

Gender	
Male	4 (25)
Female	12 (75)
Age Category	
50-54	4 (25)
55-64	8 (50)
65-74	3 (18)
75-84	1 (7)
Race	
White	12 (75)
Black	3 (18)
Prefer not to answer	1 (7)
Education	
High School	1 (7)
Some College	3 (18)
Associates	2 (12)
Bachelors	5 (31)
Graduate Degree	3 (18)
Doctorate	2 (12)
Insurance Type	
Private	11 (70)
Public	4 (25)
Missing	1 (7)
Employment Status	
Employed	8 (50)
Retired	6 (37)
Unable to Work	1 (7)
No Response	1 (7)
Length of Time Living with Pain	
Six months to less than 1 year	1 (7)
1 year to 5 years	6 (37)
More than 5 years	9 (56)
Income	
Prefer not to answer	3 (18)
$10,000 to $24,999	3 (18)
$25,000 to $49,999	3 (18)
$50,000 to $74,999	3 (18)
$75,000 to $99,999	1 (7)
$150,000 and higher	1 (7)
Missing	2 (12)

Most participants identified as White (n=12, 75%), female (n=12, 75%), and reported their age between 55-64 years old (n=8, 50%). Majority of participants’ education was an associate’s degree or higher (n=15, 93%) and reported private medical insurance coverage (n=11, 70%). Sessions lasted between 30-90 minutes. Participants described attitudes on integrative medicine, attitudes on opioids/analgesics, educational needs, and preferences, and patient’s view of relationship with provider/clinician, as summarized below. Table 3 presents themes and a sample of representative quotations.

**Table 3:** Themes and Select Representative Patient Quotations

Themes	Sub-themes	Representative Quotation
Integrative Pain Management	Integrative pain management experiences	“…[acupuncture] treatments would get me to a balanced emotional state as well as a balanced physical state.” “I've been to physical therapy two or three times over the last couple of years and that helps a little." “…sometimes [acupuncture] was helpful, but most of the time it was not…” “It [diet] is something that I'm very interested in…learning what foods are inflammatory and what foods cause inflammation.”
	Long-term use of integrative pain management	“I’m constantly seeking and I would have to say of the things you’ve listed, I've tried all of them in my, my long quest to live with less pain."
	Education on integrative pain management	"So I always find, you know, the more you can learn about something, you know, the broader your experience, then the better you can help yourself than somebody else.” “I feel like a number and they to rush you in and rush you out” and “I did not feel heard. I did not feel like a partner in my own healthcare.” “So he (physician) sent her to physical therapy and said well, this is as much as we can do. She (patient) said, Well, I will try this. You know, I'll try physical therapy first. But if that doesn't help, I want you to know, I want to see if there's other options available.” ”But now I have now gone from pain in my hands. And a lot of those (PT) exercises, I need to grip the pulleys and do all this I can't do because now my hands are in pain.”
	Barriers to integrative pain management	“I think for the most part, I mean, I have access to everything and just be like it just time management. And you know, how much does it cost?” “I have a huge resentment against technology. And I know it brings us a lot of things that are very positive and helpful. But it frustrates me. And it frustrates me for the bigger for bigger reasons other than I can't navigate it. Anyway, I have strong feelings about that. And I don't know, I wish there was an easier way to so that I could learn how not to forget about the resources that are presented to me.” “I attended the webinar to deepen my understanding, on ways to continue to take care of myself and manage pain without, you know, needing outside support without having to have a doctor visit.” “Because I need support on that financial support, you know, and or approval to to massage therapy, myofascial release, these things are not covered, they're all out of pocket expenses." “And I know because a COVID Things stink now. Socialization is an important part of pain control. And I really struggled with that, you know, there's not that many in person, things (educational events) that are safe to do now.”
Opioid and Non-opioid Analgesic Perceptions	Limit pharmacologic pain management	“Just give us some alternatives or… alternative things we can do other than the stuff we know. Because you can't always get a shot, the steroids deteriorate your body, the pain management, you know, pills or Aleve or anti-inflammatories don't always work is temporary, you know, so there's got to be other you know, even relaxation, breathing, deep, meditating, all that can help you.”
	Prescription opioid pain management	“And from what I can tell from this group, we all have the same experience not wanting to go on the opiates. That's just not a solution, long term. And yet that seems to be a lot of what the pain management people push plus the injection…” and “I have very strong feelings against narcotics, would not even consider anything in the opioid department” and “I have hydrocodone that they gave me for the pain. And I've tried really hard not to take a lot of that. I don't like the way it makes me feel. And I also get really constipated from it.”
	Personal/familial experiences with prescription opioids	“I became extremely nauseated. I wouldn't eat, couldn't eat. And during a period of two weeks, I'd lost 25 pounds within three weeks.” and “I managed to get myself off the opioids, because before that [opioid epidemic] even became an issue, I knew that my pain medicine was making my pain worse. And I am always researching and I got into all that and saw the sign of hyper analgesia.” “I've already have made them [family] aware of, you know, my health issues, and why I take you know, the medication [prescription opioids], as they have seen it in person for themselves, because I was bed ridden at one time. So they can see the difference in between how I used to be, and the things that I can do for myself now.”
	Health care experiences	“…just wants to throw a pill at it [pain]. “…it seemed like for a long, long time, everything was a fight with her [physician], and I did not feel heard. I did not feel like I was a partner in my own health care. And I didn't believe that the things [opioid medication] that she was recommending, or ordering, were the right things for me.” “Obviously, if the pain were so bad, that that was the only option then I would have to consider it but it would be frightening to be truthful.” "I want to be able to have a life, I don't want to just exist from day to day. If I get addicted to those things, the pain may be better, but there's going to be other problems.”
Educational Needs and Preferences	Knowledge is power	“Information is the name of the game” “the more information that an individual has, the better they are to address their situation.” “As I said, prior, my health is a priority to me. And so if I can learn anything, I'm very eager to read, listen, learn and open for anything new.”
	Educational session setting	“Having it online, I can look at it at any time that fits into my schedule. And like I said, that's my learning tool, especially with COVID, I can't be out and about and get sick.” “I'm able to drive and willing to, I mean [the city] is pretty big, but I'll drive just about anywhere. So I really don't have any concrete barriers. And obviously, I would take an in person, thing over a virtual occurrence, just because it's in person” “I think especially in today's day and age, and because of the pandemic, having something at your fingertips is so valuable.” “So, I really like in-person interaction. You know, even though I'm talking to a screen now, I'm talking to you. So, you know, that's helpful.” “…sometimes like listening to this is more helpful to me than just reading dry articles, hearing other people's experiences, what works, what doesn't, because in situations like this, you can have interaction and ask questions, where when you're just reading, you're sort of like stuck…” “I am trying it out the plate portion [MyPlate], it kind of puts everything in the right perspective, just looking at it, seeing it.” “when you're not feeling well, I'm not so sure I have big incentive to go online and struggle with my laptop to find an answer because everything is like a rabbit hole to me” “It’s the internet in general, the way that content is provided to us unfortunately, it yields more information than you need.”
Participant View of Relationship with Provider	Views on patient-provider relationship	“But it all just seems like they're tunnel vision focused. Okay, this is the problem. This is the pill. Give me all the options." “my goals for managing the pain is just … working with my doctors and trying to find a way because I don't want to be on opioids.” “Well, their reaction is, is rushed, they're always in a hurry. It's a short answer, with little to no explanation.”
	Barriers to patient-provider relationship	” I'm experiencing a lot of cracks and gaps in system as far is all of my doctors communicating with one another, I have my neurosurgery, I have my pain management, my primary care, I'm supposed to have an orthopedic.” “I have to go to my PCP once a month, I have to go to pain management once a month in to neuro back and forth. And none of them ever know what is going on with me...it's just the system is so broken up in referrals and missing, I just find myself, it's it's difficult to navigate the system." “I have a primary care physician. I had been seeing one before I retired. And after I went to Medicare, I could no longer see her because her practice didn't take Medicare."
Healthcare Systems	Barriers in the Health Care System	“We [patient and provider] met only after the system had dragged me around and wasted a lot of time. I would say it was about a year with pain before I was finally referred to a neurologist, and then the intake appointment with him.” “I wasn't able to see her, but probably would have addressed a few more things had I been in the office, but because it was the Medicare wellness, they annual wellness, she was somewhat limited to the things that she could do, because Medicare so specific on what you can do when their annual wellness exam. “ “you got to sit on the phone and try to hook the office people at the doctor's office up with the insurance people get the you know, you're like the man in the middle trying to get everybody to do her job, you know, and you're just sitting back waiting. And in pain, you know, it's very frustrating."

Theme 1: Integrative pain management

Integrative Pain Management Experiences

Participants were open to exploring integrative pain management options, such as acupuncture, aromatherapy, exercise, physical therapy (PT), yoga and nutrition. All participants reported trying at least one integrative pain management option; however, there was variable success in achieving pain relief. The familiarity and affinity for various options was highly varied.

Long-Term Use of Integrative Options for Pain Management

Participants discussed integrative options as a long-term approach rather than a short-term medical intervention. Participants voiced that managing their pain, overall health and well-being required an investment over time.

Education on Integrative Pain Management

Participants expressed a need for targeted education on integrative medicine, in which their healthcare provider directs them to specific interventions for their pain. A few participants expressed that when they visit with their providers during a traditional office visit, they are not receiving adequate education and resources to understand their options when it comes to integrative pain management. Participants also expected their healthcare team to provide alternative strategies when current integrative treatments were no longer suitable or sustainable if their condition had changed.

Barriers to Integrative Pain Management

Participants discussed barriers to accessing integrative pain management options such as time limitations, lack of knowledge, navigating online health information, insurance coverage and the COVID-19 pandemic. However, the consensus was that integrative options were worth learning about and pursuing.

Theme 2: Opioid and non-opioid analgesic perceptions

Limit Pharmacologic Pain Management

Most participants expressed a preference to limit or eliminate pharmacological options. This preference was particularly desired in those acknowledging that their pain may likely increase as they age.

Prescription Opioid Pain Management

When discussing opioids and other analgesic options, generally, participants were averse to opioid use and expressed a desire to use opioids as their last option for pain management.

Personal/Familial Experiences With Prescription Opioids

Participants described both negative and positive experiences with prescription opioids. Negative experiences included adverse effects such as nausea, hallucinations, and constipation. In more serious cases, this also included opioid use disorder and overdose. A small cohort of patients who reported daily opioid use and had a more positive outlook on opioids to avoid pain and maintain function shared positive experiences.

Healthcare Experiences

Participants voiced concerns surrounding use of opioids including impacts regarding the patient-provider relationship, which increased overall frustration.

For participants who were looking to avoid opioid use, several noted that they were fearful of possible dependence and addiction.

Theme 3: Educational needs and preferences

Knowledge Is Power

All participants indicated a desire to expand their knowledge about options for integrative pain management and options to improve their quality of life.

Educational Session Setting

There was a preference for an informal environment where patients could hear from experts who were well-versed in the pain management topics and a desire for resources for further exploration was highly emphasized. With the advent of COVID-19, participants were accustomed to attending virtual classes and embraced virtual events.

Some participants were still receptive to in-person meetings. Participants also desired open access information, rather than being bound to a designated event time, and felt confident in their ability to review the information on their own. Participants were also open to demonstration-based classes where they could participate virtually with an instructor in a specified activity. A social component, either through discussion or through a question-and-answer element, was also highly desired. Participants desired topic-focused materials and resources that could be printed at home or mailed to reinforce information learned during virtual classes. Participants expressed frustration with locating reputable health information due to the overwhelming amount of health information to decipher online.

Theme 4: Participant view of relationship with provider

Views on Patient-Provider Relationship

A recurring theme through the coding process was the patient-provider relationship and its role in pain management. There was mixed consensus among older adults around this relationship with various primary and specialty care providers. While many felt they were receiving good and necessary care, none had a recollection of a provider discussing integrative pain management options with them. As previously mentioned in the subtheme prescription opioid pain management, some participants also said medication options were recommended even if they preferred not to take medications to manage their pain. The continuous theme was a desire to approach care “as naturally as possible.” There was discussion surrounding the importance of following a doctor’s recommendations but also advocating for one’s personal preferences. There was significant frustration expressed when some older adults felt like they had exhausted all resources or did not get the answers that they were hoping for.

Barriers to Patient-Provider Relationship

Perceived barriers to an effective patient-provider relationship included limited time, communication gaps, inconsistent messaging among providers, and accessibility.

Theme 5: Healthcare systems

Barriers in the Healthcare System

Many expressed frustrations with the healthcare system as a whole, particularly with referral coordination and third-party payer restrictions as well as provider communication between specialties. Participants provided personal accounts of the difficulties in accessing specialists, obtaining lab tests and/or imaging and scheduling procedures due to the perceived inability of providers to share documentation.

## Discussion

This study explored older adult views on integrative pain management, specifically their educational needs and preferences, to inform effective strategies for patient-centered pain management education. Participants provided insight into their openness to integrative pain management, the importance of long-term use of integrative medicine, their desire for more education on integrative approaches, barriers to obtaining integrative medicine, and their preference to limit pharmacologic pain management. Participants also shared their perception of opioid pain management, personal and familial experiences with prescription opioids, experiences with the healthcare system, their educational needs and preferences on integrative medicine and an in-depth view of the patient-provider relationship. 

Although participants had varied levels of knowledge and experience with integrative pain management, contrary to previous reports describing an unfamiliarity with opioid alternatives, all older adult participants reported having tried at least one non-pharmacologic modality to manage their pain [9]. All participants desired education and guidance from healthcare providers on: integrative options tailored to address their specific pain condition; how to best access these options (find reputable licensed practitioners, navigate insurance and cost); and alternative strategies when current integrative treatments were no longer suitable or sustainable.

Unmet educational needs were an important theme discussed by participants. Studies have previously demonstrated the benefits of education in improving pain outcomes but, to our knowledge, have not fully explored patient preferences in how pain education should be delivered [12,20]. Our participants identified insufficient time during a traditional clinical appointment as a barrier to fulfilling their educational needs on integrative pain management options. They preferred informal, non-clinical environments to receive education and instruction from various health experts. Most favored on-demand open-access internet-based or virtual formats to schedule in-person events and were confident in their ability to review provided resources and information. Participants cited a virtual format as more convenient than in-person events as it eliminated transportation and mobility barriers. Additionally, because of the COVID-19 pandemic, many expressed a preference for virtual formats that incorporated demonstrations and opportunities for audience interaction with presenters. This perceived convenience expressed by participants is supported by literature noting similar barriers to older adults’ participation in in-person educational events. These barriers include inability to travel, lack of transportation, time conflicts, and a preference to avoid group activities [21-23]. Additionally, virtual pain management programs have demonstrated comparable pain outcomes to traditional formats, even in older adults [23]. While virtual formats were favored by most, a minority of participants preferred in-person settings to receive education.

Participants recognized that the COVID-19 pandemic led to sweeping changes in social interactions and environments in and outside of the healthcare setting, limiting their access to pain management therapies, education and guidance provided by pain management providers. Social isolation is well linked to negative physical and mental health consequences and may disproportionately impact older adults living with chronic pain [24,25]. Promising strategies to mitigate the negative consequences resulting from COVID-19 social distancing in chronic pain patients have been reported [24,25]. Although participants reported favoring virtual educational programs and materials, they also noted they would prefer virtual programs to incorporate social components, such as live and interactive discussions. Thus, pain management educational programs should offer opportunities for social connectedness to address these social needs.

While study participants preferred a virtual format, they also desired physical materials and handouts when feasible. As the link between health literacy and health outcomes is well established, developing patient-centered educational materials and programs is essential for any educational strategy to be effective and result in improved pain outcomes [7].

Participants held divergent views on the usefulness of prescription opioids in managing their pain, with some reporting opioid-induced side effects interfered with their ability to perform daily activities and others noting prescription opioids improved their function and pain. Most were concerned with adverse effects and risk for addiction. Conflicting patient views on the use of prescription opioids for chronic pain have been described previously in the literature, with one study noting patients with a positive view on opioids had developed coping strategies to deal with their fears of addiction and felt supported by their physician [26]. Consistent with the literature, our participants expressed use of prescription opioids often impacted the patient-provider relationship, noting open dialogue and improved education were needed on opioid alternatives and risks to address patients’ fear of opioid medications [21].

Several codes related to the patient-provider relationship emerged, however only those relevant to our study objectives were presented and discussed. Treatment success, compliance and satisfaction with pain management have previously been linked with the quality of the patient-provider relationship [27]. The study team identified several barriers to the patient-provider relationship including limited time, communication gaps, and accessibility. Similar obstacles to the patient-provider relationship have been described previously [21,27-29]. It is clear that improved methods or strategies for effective communication are needed to enhance the traditional patient-provider encounter. Strategies for improvement may include providing healthcare providers specific trainings focused on patient-centered communication techniques and pain management goal development, utilizing other healthcare staff to assist with patient education and counseling, such as a health educator, and providing resources and materials to patients that are tailored to their health literacy level. Future in-depth studies are warranted to further explore the patient-provider relationship and identify effective communication strategies in the context of pain management. 

Limitations

There are several important limitations to note. First, as recruitment was confined to one geographic region and limited by self-selection bias, perspectives expressed by participants may not represent those of populations residing in other geographic regions, those who are non-English speaking, or even those living within the region who elected not to participate. Furthermore, this study required virtual participation, thus may have had limited perspectives from less technologically literate or low-income populations. Additionally, the sample interviewed had high education levels, low representation from males and non-white individuals. We also did not collect specific pain characteristics for participants (type of pain, pain severity, etc). To ensure participants met study eligibility, study inclusion criteria were reviewed with participants prior to enrollment. Lastly, the electronic consent process and virtual format of the focus group and interviews may have limited participation.

## Conclusions

This study provides novel insights on older adults’ perceptions of integrative pain management, as well as their educational needs and preferences on this topic. These preferences should be confirmed in other older adult pain populations, particularly minority and low technology access populations. Participants appear receptive to the concept of integrative pain management, recognizing its potential to increase self-management and self-efficacy, limit analgesic and opioid use, and offer a sustainable long-term pain management approach compared to traditional pharmacologic and opioid-centric approaches. Virtual pain management educational initiatives incorporating live demonstrations, audience participation and interaction, and physical resources and materials may be preferable to older adults compared to traditional in-person or exclusively recorded, asynchronous formats. Future studies are needed to address the implementation of such educational interventions.

In this study, participants clearly expressed their educational needs related to integrative pain practices are not being met through traditional healthcare interactions and this perceived gap is a barrier in their ability to incorporate these practices into their lives. Our study findings related to educational preferences can potentially be used to develop patient-centered educational programs to overcome this barrier. Future studies utilizing quantitative methods can determine whether such programs may improve the quality of care and life of aging adults living with pain through promotion of education and self-efficacy, using person-centered, multidisciplinary, and integrative approaches for pain management.

## Appendices

**Table 4:** Research Participant Discussion Guide Credits: Sophia Sheikh, Jennifer Brailsford, and Nicole Spindle

Introduction Question
What are your goals in terms of pain and its impact on your life?
Needs assessment
What do you feel is not working in your pain management plan?
What do you feel is working well?
Do you feel your caregiver/family understands how pain is impacting your life?
What are your thoughts on communication between yourself and your pain management provider? How can it be improved? What is good about it?
Do you feel they understand how much pain is impacting your life?
Do they listen to you while you talk about your pain?
How does time factor into your ability to discuss your pain with your provider?
Do you believe you play a role in developing the pain management plan set by your provider?
Do you believe the success of your pain management plan depends, in part, on you?
Do you sometimes forget what you want to tell your provider regarding your pain?
What are your thoughts on using a pain app or pain journal to track your pain over time?
Attitudes/beliefs
Do you feel that you are judged differently if you use opioids to treat your pain?
Have you ever experienced anxiety, sadness, or hopelessness regarding your pain?
If so, have you expressed these feelings with your loved ones or doctor?
Do you currently have any non-medication pain management strategies that you use?
What are your thoughts on pain medications other than opioids for treating your pain?
What are your experiences with using approaches other than pain medications to treat pain?
Has your provider talked to you about options other than opioids for pain?
What factors do you think may limit your ability to use these approaches? (money, time, etc)
Do you worry that increasing your activity level will worsen your pain?
Have you used a combination of opioids and non-medication treatments together to treat your pain?
What are your thoughts on combining opioids and non-opioid treatment options together? How about pain medications and techniques other than medications to treat pain?
Perspectives on Pain Management Education
What are your thoughts on attending a workshop on how to manage pain using non-medication approaches? Approaches such as… Virtual reality Relaxation techniques Mindfulness Meditation Aromatherapy Exercise Physical therapy Acupuncture
What would you hope to learn from this workshop?
What elements of the workshop would keep you returning to other workshops?
How do you best learn?
What length of time would you find suitable for a workshop?
Do you enjoy classes that include discussion?
Do you prefer to observe an activity before you try it?
Would you be willing to participate in physical activity at a workshop if you were instructed?
Would you prefer to receive take-home materials to further explore workshop topics at home?
Are there other barriers that would prevent you from attending a workshop (physical location; stairs; navigating building)?
What topics in pain management would you like to learn more about in a workshop setting? Virtual reality Relaxation techniques Mindfulness Meditation Aromatherapy Exercise Physical therapy Acupuncture
Concluding question
Of all the things we’ve discussed today, what do you think is the most important aspect for us to address in the workshop?
